# Higher Mallampati Scores Are Not Associated with More Adverse Events During Pediatric Procedural Sedation and Analgesia

**DOI:** 10.5811/westjem.2017.11.35913

**Published:** 2018-02-26

**Authors:** Maya S. Iyer, Raymond D. Pitetti, Melissa Vitale

**Affiliations:** * The Ohio State University College of Medicine/Nationwide Children’s Hospital, Department of Pediatrics, Section of Emergency Medicine, Columbus, Ohio; † Children’s Hospital of Pittsburgh of UPMC, Department of Pediatrics, Division of Pediatric Emergency Medicine, Pittsburgh, Pennsylvania

## Abstract

**Introduction:**

Procedural sedation and analgesia (PSA) is used by non-anesthesiologists (NAs) outside of the operating room for several types of procedures. Adverse events during pediatric PSA that pose the most risk to patient safety involve airway compromise. Higher Mallampati scores may indirectly indicate children at risk for airway compromise. Medical governing bodies have proposed guidelines for PSA performed by NAs, but these recommendations rarely suggest using Mallampati scores in pre-PSA evaluations. Our objective was to compare rates of adverse events during pediatric PSA in children with Mallampati scores of III/IV vs. scores of Mallampati I/II.

**Methods:**

This was a prospective, observational study. Children 18 years of age and under who presented to the pediatric emergency department (PED) and required PSA were enrolled. We obtained Mallampati scores as part of pre-PSA assessments. We defined adverse events as oxygen desaturation < 90%, apnea, laryngospasm, bag-valve-mask ventilation performed, repositioning of patient, emesis, and “other.” We used chi-square analysis to compare rates of adverse events between groups.

**Results:**

We enrolled 575 patients. The median age of the patients was 6.0 years (interquartile range = 3.1,9.9). The primary reasons for PSA was fracture reduction (n=265, 46.1%). Most sedations involved the use of ketamine (n= 568, 98.8%). Patients with Mallampati scores of III/IV were more likely to need repositioning compared to those with Mallampati scores of I/II (p=0.049). Overall, patients with Mallampati III/IV scores did not experience a higher proportion of adverse events compared to those with Mallampati scores of I/II. The relative risk of any adverse event in patients with Mallampati scores of III/IV (40 [23.8%]) compared to patients with Mallampati scores of I/II (53 [18.3%]) was 1.3 (95% confidence interval [0.91–1.87]).

**Conclusion:**

Patients with Mallampati scores of III/IV vs. Mallampati scores of I/II are not at an increased risk of adverse events during pediatric PSA. However, patients with Mallampati III/IV scores showed an increased need for repositioning, suggesting that the sedating physician should be vigilant when performing PSA in children with higher Mallampati scores.

## INTRODUCTION

Procedural sedation and analgesia (PSA) is the use of sedative, analgesic, or dissociative drugs to relieve pain and anxiety associated with diagnostic and therapeutic procedures, while maintaining continuous and independent ventilation without loss of protective reflexes.^1^, ^2^ Many procedures that were formerly performed under general anesthesia in the operating room (OR) are now successfully completed using PSA in locations outside the OR, including the emergency department (ED). 3 As a result, PSAs are now being performed more frequently by non-anesthesiologists (NAs), such as emergency physicians, and it is estimated that roughly a quarter of a million pediatric patients will receive PSA in the ED alone each year.^4^, ^5^ Therefore, it is paramount that emergency physicians be prepared not only to administer proper PSA to children, but also to manage any complications or adverse events that may arise when PSA takes place in the pediatric ED.

Adverse event rates during pediatric PSA in non-OR settings are reported between 2.3%-17.6%.^6^, ^7^ The definition of adverse events during PSA varies in the literature and has included the following: oxygen desaturation less than 90–93%; apnea; stridor; laryngospasm; bronchospasm; cardiovascular instability; paradoxical reactions; emergence reactions; emesis; and aspiration.^5^, ^6^ Of these, the adverse events that pose the most significant risk to the safety of the patient are those that compromise the airway.^8^ Smaller studies have found rates of airway compromise during PSA ranging from 5–6%.^9^–^11^ Medications used for PSA varied in these studies and included chloral hydrate, propofol, ketamine, midazolam and fentanyl.

Larger studies have also found significant but lower rates of airway compromise among pediatric patients undergoing PSA. The Pediatric Sedation Research Consortium found that among nearly 30,000 PSAs performed outside the OR, oxygen desaturation occurred 157 times per 10,000 sedations; stridor and laryngospasm both occurred in 4.3 per 10,000 sedations; and unexpected apnea occurred in 24 per 10,000 sedations.^12^ Finally, similar adverse event rates with oxygen desaturations were reported in a systematic meta-analysis of studies involving PSA in the ED.^13^ Thus, evidence shows that despite various medications used in pediatric PSAs in different settings, the risk of airway compromise remains.

Because of the universal risk for airway compromise among PSA medications, further research has sought to identify patient factors that predict higher risk for adverse events during PSA. For example, studies have shown that patients of younger age (<1–2 years old) or with higher American Society of Anesthesiologists (ASA) classification (ASA>2) may experience more adverse respiratory events during PSA.^9^, ^14^, ^15^ In light of the inherent airway risks of PSA and the potential ability to identify predisposing factors for adverse events prior to PSA, professional medical governing bodies have proposed guidelines and recommendations specifically for PSA performed by NAs. The guidelines encompass risk assessment prior to PSA by performing a complete history and physical exam and determining ASA classification and nil per os (NPO) status. They also stress the importance of appropriate monitoring during PSA and access to airway rescue equipment and pharmacological reversal agents. When implemented, these guidelines have proven to decrease the rate of respiratory adverse events.^16^–^19^

It is notable, however, that the guidelines cursorily, if at all, suggest using Mallampati scores in pre-PSA evaluations. Mallampati scores are obtained by visualizing a patient’s posterior oropharynx while the patient is seated and opening his mouth with his tongue protruded. The modified Mallampati classification scheme scores adequacy of visualization from I to IV, with I being full visualization and IV being visualization of only the hard palate. The Mallampati score is used to predict difficulty with intubation, with those who score III or IV being more difficult to intubate, and has been validated in children.^20^–^23^ Given that a higher Mallampati score may indirectly indicate children who have potentially difficult or anatomically different airways, this classification scheme may add important risk information to pediatric pre-PSA assessments. Thus, the objective of this study was to assess whether pre-PSA Mallampati score can predict adverse events during pediatric PSA.

**Population Health Research Capsule**

What do we already know about this issue?

While pediatric procedural sedation and analgesia (PSA) is considered safe, the most common adverse events are related to respiratory compromise.

What was the research question?

Would children with higher Mallampati scores experience higher rates of adverse events during PSA?

What was the major finding of the study?

Patients with higher and lower Mallampati scores had similar adverse event rates with the exception of need for repositioning.

How does this improve population health?

Because many non-pediatric physicians perform PSA, this study reinforces that vigilant monitoring during PSA is necessary to provide optimal care for our pediatric patients.

## METHODS

### Study Design

This was a prospective observational study that took place between March – August 2013 at a tertiary care institution that performs approximately 900 PSAs in the PED annually. This study received institutional review board approval from the participating institution.

### Study Setting and Population

We included all children between the ages of 0 and 18 years who presented to the PED for medical or surgical conditions requiring procedural sedation during the study period. Children with genetic syndromes, anatomic abnormalities such as retrognathia, cleft lip and/or palate, macroglossia or any other medical condition that would preclude PSA were automatically excluded since they did not meet criteria for PSA in the PED.

### Study Protocol

#### Mallampati Scoring and Documentation

Sedations were performed by pediatric emergency medicine (PEM) attending physicians, PEM fellows, or senior pediatric or EM residents on their sedation rotation in the PED. The sedating physician was responsible for performing a pre-PSA evaluation, including obtaining a Mallampati score, which was documented in the patient’s electronic medical record (EMR). Given that the Mallampati scoring system has previously been shown to have moderate inter-rater reliability and that having multiple physicians perform a pre-PSA assessment would hinder the workflow in the PED, only one physician obtained the Mallampati score for each patient.^24^ The modified Mallampati scoring system is illustrated in the figure1.^20^, ^25^

Prior to the study period, senior pediatric and EM resident physicians performing PSAs participated in a four-hour procedural sedation course at the beginning of their academic year and received a follow-up, 30-minute, one-on-one sedation simulation session prior to their sedation rotation in the PED. During both of these sessions, residents received verbal and written instructions on how to use the Mallampati scoring system and how to document these scores in the EMR. PEM attending physicians and fellows received both verbal and written instructions on Mallampati scoring and documentation during two separate sessions. Finally, all physicians completing pre-PSA assessments received a pocket card outlining the Mallampati scoring so that it could be referred to at the patient’s bedside.

#### Definition of Adverse Events

Given that the adverse events posing the most significant risk to the safety of the patient are those that compromise the airway, the following were categorized as adverse events for the purposes of this study: oxygen desaturation < 90%; apnea as defined of cessation of spontaneous ventilation for 20 seconds; laryngospasm; need for bag-mask ventilation (BMV) as determined by the physician performing PSA; emesis; and events categorized as “other” by the physician administering PSA.^7^ Additionally, the need for repositioning was also considered to be an adverse event (though of less significance than the aforementioned) for the purposes of this study, since children with larger occiputs could be more prone to obstructive airway compromise.

### Data Collection

In addition to adverse events, we collected the following data points for this study: past medical history; past surgical history; allergies; Mallampati score; ASA classification; previous anesthesia; reason for sedation; medication(s) given during sedation; vital signs; use of suctioning; use of supplemental oxygen; length of sedation; length of procedure; and total length of stay in the ED. The data was stored in the Research Electronic Data Capture (REDCap^TM^, Vanderbilt University, 2013), an online software that allows for database creation and statistical analysis.

### Data Analysis

Based on previous studies, we estimated that 38% of the general population would have a Mallampati score of III or IV.^26^ The known adverse event rate for PSA at the site of this study is approximately 10%. Based on these assumptions, and in order to detect a 15% difference in the rate of adverse events between individuals with Mallampati scores of III/IV vs. those with Mallampati scores of I/ II, a minimum of 204 study subjects (n= 125 for Mallampati III/IV and n=79 for Mallampati I/II) were required for enrollment (α= 0.05 and β= 0.20).

We used descriptive statistics to analyze patient characteristics and demographic data. For categorical data, groups were compared using chi square or Fisher’s exact test, where appropriate. The Kruskal-Wallis test was used to compare patient characteristics between the Mallampati groups. We performed all statistical analyses using SPSS Statistics for Windows Version 24 (IBM Inc., Armonk, NY).

## RESULTS

During the study period 575 PSAs were completed. Mallampati scores were documented in 458 patients, and were either not recorded or unable to be obtained secondary to patient compliance in 117 patients. The median age of the patients undergoing PSA was 6.0 years (interquartile range [IQR] 3.1, 9.9). The majority of patients in the study population who underwent PSA were Caucasian (n= 461, 80.2%) and male (n=327, 56.9%). The median length of sedation was 24.0 minutes (IQR=17.0, 32.0). The primary reasons for sedation were fracture reduction (n=265, 46.1%) and laceration repair (n= 153, 26.6%). In addition, 92.5% (n=532) of the patients were categorized as ASA I or II. The majority of sedations used ketamine either alone or in combination with another medication (n= 568, 98.8%). Table 1 illustrates these characteristics based on Mallampati scores.

Table 2 shows the number of adverse events during PSA by Mallampati scores. Patients with Mallampati scores of III/IV did not experience a significantly higher proportion of adverse events compared to those with Mallampati scores of I/II. However, a higher proportion of those with Mallampati scores of III/IV compared to those with Mallampati scores of I/II required repositioning during PSA (p < 0.05). The relative risk of any adverse event in patients with Mallampati scores of III/IV [40 (23.8%)] compared to patients with Mallampati scores of I/II [53 (18.3%)] was 1.3 (95% confidence interval [CI] [0.91–1.87]).

## DISCUSSION

Our study found that there was not a significant difference in the proportion of adverse events between those individuals with Mallampati scores of III/IV vs. those with Mallampati scores of I/II. In fact, post-hoc power analysis showed that this study had a 95% power to detect a 15% difference in the proportion of adverse events between these two groups. Notably, a greater proportion of patients with Mallampati scores of III/IV compared with those of Mallampati scores of I/II required repositioning during PSA. This is not surprising since those with higher Mallampati scores likely have the body habitus, particularly increased neck girth and larger facies, that could predispose a patient to obstructive respiratory events during PSA. Hirsch et al. found that children who were obese and undergoing PSA experienced a greater desaturation rate compared with children who were not obese (9.9% vs 5.4%; p=0.04).^27^ Furthermore, Mallampati scores have also been shown to be an independent predictor of obstructive sleep apnea, thus highlighting the fact that these scores may be an indirect measurement of anatomical factors that should be considered in pre-PSA assessments.^28^

In this study, nearly 20% of the patients did not have a documented Mallampati score or the physician administering PSA was unable to obtain a score. Although the physicians who did not document a Mallampati score were not required to provide information on why these scores were not reported, we surmise that the primary reason for scores not obtained was secondary to patient compliance. The median age of those whose scores were either not obtained or unable to be obtained was 2.6 years, thus suggesting that age may limit the physician’s ability to obtain a Mallampati score. Mallampati scoring requires the patient to sit upright, voluntarily open his mouth and refrain from saying “ahh” (a maneuver that falsely elevates the palate). Koop et al. showed that children under the age of four are less likely to be able to cooperate with such maneuvers and may not have the cognitive ability to follow through with multi-step tasks that require greater attention.^29^, ^30^

Similarly, other studies comparing Mallampati scores to other indirect methods predicting difficult endotracheal intubations, such as the Cormack and Lehane grading system, have also encountered difficulty in obtaining Mallampati scores for children ages 1–3 years.^31^, ^32^ Furthermore, pediatric patients presenting to the PED for PSA are often suffering from painful injuries, and under these circumstances physical examinations, particularly oropharyngeal exams, can be viewed as distressing from the patient and parent perspectives.^33^ Thus, age, physical pain, and distress or anxiety may hinder the physician’s ability to obtain a Mallampati score.

While this study was powered to detect differences in adverse events between those with Mallampati scores of III/IV vs. those with Mallampati scores of I/II, it is still rare for adverse, sedation-related events to occur, particularly with the use of ketamine. The adverse event rate during the study period was 11.6%, which is similar to previously reported PSA adverse event rates at this institution.^8^, ^15^ This event rate is, however, higher than the reported adverse event rate with using ketamine as a single agent (0.4%–2.3%).^34^, ^35^ This discrepancy may be due to the fact that children in this study may have had more than one adverse event documented during a single sedation, such as apnea, oxygen desaturation, and BMV.

Previous studies have shown that ketamine has a low side-effect profile with the most common adverse events being those related to respiratory compromise and emesis.^11^, ^36^ In fact, the odds of respiratory adverse events associated with ketamine use increases when it is administered intramuscularly instead of intravenously.^36^ In addition, ketamine-associated emesis can be reduced by administering ondansetron prior to the start of PSA.^33^ However, neither of the two patients who had emesis during PSA in this study received ondansetron as a premedication. Moreover, while the authors did not evaluate NPO status and how this relates to emesis, previous studies have shown that the NPO time does not affect the rate of major adverse events during PSA.^37^, ^38^

## LIMITATIONS

There are a few limitations in this study. We did not analyze the route of administration of the PSA agent during initial data collection. Also, ketamine was the primary PSA agent in this study and if different institutions use other medications, such as an opioid or benzodiazepine, the results may vary. This, along with being a single-site study, limits the generalizability of the results. Furthermore, although adjustments for confounders were not made in this study, Table 1 illustrates that the Mallampati III/IV and Mallampati I/II groups were very similar with the covariates that were measured.

Additionally, the definitions for adverse events in this study focused on those involving airway compromise and the thresholds used were slightly different from those previously described in the literature. For instance, we used the definition of oxygen desaturation <90% to account for true hypoxia requiring supplemental oxygen instead of the definition of oxygen desaturation occurring between 90–93%. 5, 6 Consequently, the rate of oxygen desaturation may be lower in this study. Finally, since it is not routine procedure at this institution to use end-tidal CO2 during PSA, this may have limited our ability to obtain true objective data in regard to apneic episodes.

## CONCLUSION

We found that there was not a significant difference in the rate of adverse events between patients with Mallampati scores III/VI compared to those with Mallampati scores of I/II. However, patients with Mallampati scores of III/VI had a higher proportion needing repositioning, suggesting that the sedating physician should be more vigilant with these patients.

## Figures and Tables

**Figure:**
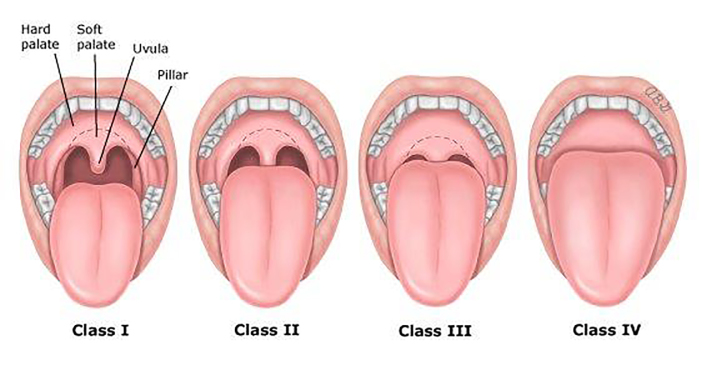
The modified Mallampati classification system. Reproduced with permission from: Walls, RM, Brown, CA. Approach to the difficult airway in adults outside the operating room. In: UpToDate, Post, TW (Ed), UpToDate, Waltham, MA. (Accessed on December 7, 2017). Copyright © 2017, UpToDate, Inc. For more information visit www.uptodate.com.

**Table 1:** Characteristics of the patients undergoing procedural sedation and analgesia, with Mallampati score.

Characteristic	Mallampati I/II n= 290	Mallampati III/IV n= 168	Mallampati not assessed/documented n= 117
Age, y, median (IQR)	7.0 (4.2, 10.9)	6.7 (3.9,10.8)	2.6 (1.6,5.0)
Male, n (%)	154 (53.1)	109 (64.9)	64 (54.7)
Caucasian race, n (%)	236 (81.4)	135 (80.4)	90 (76.9)
Length of sedation, min, median (IQR)	24 (17.0, 31.0)	25 (17.0, 34.0)	25 (16.0, 31.0)
ASA I or II, n (%)	277 (95.5)	165 (98.2)	90 (76.9)
Procedure, No. (%)			
Fracture reduction	153 (52.8)	83 (49.4)	29 (24.8)
Laceration repair	67 (23.1)	44 (26.1)	42 (35.9)
Abscess, incision and drainage	37 (12.8)	24 (14.3)	32 (27.4)
Lumbar puncture	1 (0.3)	1 (0.6)	1 (0.9)
Nailbed repair	15 (5.2)	5 (3.0)	8 (6.8)
Genital injury	5 (1.7)	1 (0.6)	0 (0)
Other	12 (4.1)	10 (6.0)	5 (4.3)
Sedation drugs, No. (%)			
Ketamine	159 (54.8)	97 (57.7)	82 (70.1)
Combination (ketamine + another medication)	129 (44.5)	68 (40.5)	33 (28.2)
Other	1 (0.3)	1 (0.6)	1 (0.9)

*ASA*, American Society of Anesthesiologist Classification System; *IQR*, interquartile range

**Table 2:** Adverse events by Mallampati score

Adverse Event Occurred n (% within Mallampati group)	Mallampati I/II n= 290	Mallampati III/IV n= 168	P-value
Any adverse event	53 (18.3)	40 (23.8)	0.16
Oxygen desaturation	20 (6.9)	16 (9.5)	0.31
Bag mask ventilation	3 (1.0)	2 (1.2)	1.00
Repositioning	19 (6.6)	20 (11.9)	0.049
Laryngospasm	3 (1.0)	3 (1.8)	0.67
Apnea	2 (0.7)	1 (0.6)	1.00
Emesis	2 (0.68)	0 (0)	0.53
Other	18 (6.2)	11(6.5)	0.89
